# A novel ROCK inhibitor: off-target effects of metformin

**DOI:** 10.3906/biy-2004-12

**Published:** 2021-02-09

**Authors:** Aysun ÖZDEMİR, Mustafa ARK

**Affiliations:** 1 Department of Pharmacology, Faculty of Pharmacy, Gazi University, Ankara Turkey

**Keywords:** Connectivity MAP, Rho kinase, metformin, off-target effect, oral antidiabetics

## Abstract

In drug discovery, most small molecules cannot cross many stages, only a few can become drug candidates. Once the drug molecule is approved and marketed, nontarget effects that are not easily distinguishable from the actual target of the drugs might be evaluated. This situation restricts the treatment. Thus, the discovery of new drugs is a very long and expensive process. In recent years, without developing new drugs, the approach of using different and new target molecules in new indications apart from the indications of licensed drug molecules has gained importance.In this study, using the connectivity map program, it was determined that metformin and tolbutamide used in the treatment of type II diabetes had the potential to inhibit Rho kinase. In the experimental results to confirm this data, it has been shown that metformin and tolbutamide decrease the cell area within 24 h and metformin inhibits the activation of Rho kinase in MCF-7 cells.These results indicate that metformin, which is used in the treatment of type II diabetes, acts as a ROCK inhibitor. Metformin has potential in the treatment of various pathological conditions in which Rho kinase has a role.

## 1. Introduction

Identifying drug targets for the treatment of diseases and developing new compounds that can induce the desired effect through drug-target interaction is a very long and expensive process for researchers. In recent years, apart from the indications for the approved drug molecules, the approach of using these drugs in different indications by identifying different and new target molecules has gained importance. Thus recently, in silico pattern-matching, software are widely used to identify new targets for small molecules.

The connectivity map (CMAP) program is a web-based library that is produced by the Broad Institute (Cambridge, MA, USA). CMAP includes 1.5 million gene expression profiles from various cell lines (A375, A549, HCC515, HEPG2, HT29, MCF7, PC3, HA1E, VCAP) that were treated with ~ 5000 small molecule compounds (Lamb et al., 2006). The software is a catalog comparing the similarities of changes at the gene expression level induced by small molecules, and scoring the similarity.

In 1996, Rho kinase (ROCK) was identified as the downstream effector of Rho A, which mediates many intracellular signaling mechanisms (Kimura et al., 1996; Nakagawa et al., 1996; Ark et al., 2010; Özdemir et al., 2016). ROCK has two different isoforms: ROCK I (p160ROCK, ROKβ) and ROCK II (ROKα) (Kimura et al., 1996; Nakagawa et al., 1996). Inhibition of Rho kinases affects the function of many downstream substrates, such as the myosin binding subunit of myosin light chain phosphatase (MLCP), myosin light chain (MLC), LIMK kinases (LIM kinases 1 and 2), myristoylated alanine-rich C-kinase (MARCKS), collapsing response mediator protein-2 (CRMP2), adducin, sodium/hydrogen exchanger 1 (NHE1) and ezrin-radixin-moesin (ERM) (Riento and Ridley, 2003). Rho kinase inhibitors have a potential therapeutic efficacy in a wide range of pathological conditions, such as asthma (Schaafsma et al., 2008), cancer (Rath and Olson, 2012), erectile dysfunction (Albersen et al., 2010), glaucoma (Olson, 2008), insulin resistance (Lee et al., 2009), kidney failure (Komers et al., 2011), neuronal degeneration (Mueller et al., 2005) and osteoporosis (Olson, 2008). However, only two ROCK inhibitors have been approved for the treatment of diseases. Fasudil, the Rho kinase inhibitor, was first approved in 1995 for the treatment of cerebral vasospasm in Japan and China (Bito et al., 2000). Ripasudil, another Rho kinase inhibitor, was approved for use in glaucoma treatment in Japan in 2014 (Garnock-Jones, 2014).

Several studies showed that oral antidiabetic drugs might have a clinical use on various diseases other than the use of type II diabetes. For instance, metformin, one of the oral antidiabetic drugs, has been reported in the treatment of polycystic ovary syndrome (PCOS) in which hyperinsulinaemia is accompanied (Diamanti et al., 2010). Additionally, metformin inhibited the proliferation of cancer cells in different cancers, such as ovarian, melanoma, prostate, and breast cancers (Saraei et al., 2019). Another group of drugs used in the treatment of diabetes are u204a (SGLT2) inhibitors. These drugs also show cardiovascular and renal protective effects through the reduction of u204d by diuretic effect and the reduction of afterload by lowering blood pressure (Lytvyn et al., 2017). These pleiotropic on-target effects pointed out that the potential off-target effect of oral antidiabetic drugs might be effective in different pathological conditions.

The aim of this study was to identify an off-target of FDA-approved oral antidiabetic drugs that might be potential candidates as ROCK inhibitor using connectivity map.

## 2. Materials and methods

### 2.1. In silico study

Connectivity map database (Lamb et al., 2006; Jun et al., 2017) was used to search the correlation between oral antidiabetic drugs and Rho kinase inhibitors. In connectivity map we used the touchstone tool which contains expression profiles of a variety of cell lines-treated with thousands of chemicals. We researched metformin and tolbutamide separately in the touchstone tool. When we detailed the list about correlation score, we chose Rho kinase inhibitors against metformin and tolbutamide. A positive connectivity score indicated that there is a similarity among the expression levels of genes induced by small molecules. In general, in order to have a strong hypothesis, the score should be 90 and above. From the list obtained, the high scores obtained in eight cell lines of Rho kinase inhibitor fasudil and ripasudil were selected. All the data obtained from CMAP were found at Broad Institute1Broad Institute (2020). Connectivity Map 02 [online]. Website http://www.broad.mit.edu/cmap/ [accessed 00 Month Year].. 

### 2.2. Cell culture

Human breast cancer MCF-7 obtained from ATCC and human cervical cancer HeLa obtained from Şap Institute (Ankara, Turkey) cell lines were used in this study. The cells were grown in DMEM (Dulbecco’s modified eagle medium) (Sigma-Aldrich Corp., St. Louis, MO, USA; cat no: D6429), containing 10 % fetal bovine serum (Sigma-Aldrich Corp.; cat no: F9664), 100 U/mL penicillin and 100 µg/mL streptomycin (Capricorn Scientific GmbH, Ebsdorfergrund, Germany; cat no: PS-B) at 37 °C. When the cells reached 70%–80 % confluency, they were counted and the specified number of cells were seeded in petri dishes.

### 2.3. xCelligence assay

The xCELLigence system real-time cell analyzer was used for the determination of nontoxic concentrations of metformin and tolbutamide (Özdemir and Ark, 2013). Briefly, this system measures electronic impedance through microelectrodes placed on the surface of special 16-well plate called e-plate. After the cells are seeded on these special plates, as the attached cell number increases, the electronic impedance increases.

10,000 cells were plated on e-plates and monitored every 15 min for 24 h. After 24 h attachment, the media was replaced with fresh media. Then cells were exposed to metformin and tolbutamide at indicated concentrations for another 24 h. Since there was a solubility problem at concentrations above 300 µmol, we tested drugs with concentrations starting at 300 µmol and decreasing logarithmically. The slopes of the curves were calculated using the software of the xCELLigence RTCA DP system as an indicator of cell proliferation.

### 2.4. Holographic 3D cell analysis

HeLa and MCF-7 cells were cultured in 35-mm dish (Ibidi GmbH, Gräfelfing, Germany; cat no: 81156) and exposed to 100 µM and 300 µM tolbutamide (Sigma-Aldrich Corp.; cat no: T0891) and metformin (as a gift from Bilim İlaç San. ve Tic. A.Ş., Kocaeli, Turkey) for 24 h. The images holographic of the cells were captured randomly by HoloMonitor M4 instrument (Phase Holographic Imaging PHI AB, Lund, Sweden) at 1 h and 24 h. Alteration of the cell morphology was analyzed by HoloStudio 2.4 software (Phase Holographic Imaging PHI AB). 

### 2.5. Activation determination of cofilin (phospho-Ser3)

Phosphorylation of cofilin was performed using phospho-cofilin (Ser3) colorimetric cell-based ELISA kit (Aviva Systems Biology Corp., San Diego, CA, USA; cat no: OKAG01911) according to the manufacturer’s instructions (Şimay et al., 2018). Ten thousand HeLa and MCF-7 cells were seeded to each well of the kit. Then, the next day, the cells were incubated with metformin and tolbutamide for 1 h. The cells were then fixed for 20 min at room temperature. After washing the plate with wash buffer three times, blocking buffer was added to each well and the plate was incubated for 1 h at room temperature. The plate was washed three times. Then antibodies for the target antigen (cofilin, phospho-cofilin, GAPDH) were added to each well and cells were incubated for 16 h at 4 °C. After each well was washed, the cells were incubated with HRP-conjugated secondary antibodies for 2 h at room temperature with gentle shaking. Following washing the plate three times, TMB substrate was added and the plate was incubated for 30 min at room temperature. Then the stop solution was added to each well. The absorbance of each well was read at 450 nm immediately.

### 2.6. Statistical analysis

All the values were assessed using one way ANOVA test followed by Tukey’s post hoc test to analyze multiple group comparison using GraphPad Prism version 6.0 (GraphPad Software Inc., La Jolla, CA, USA). Statistical significance was set at P ≤ 0.05.

## 3. Results

### 3.1. Analysis of ROCK inhibitor candidates using connectivity map

To explore whether metformin and tolbutamide have a possible inhibitor effect on Rho kinase, we utilized the CMAP by comparing the changes in gene expression between metformin and tolbutamide, and various Rho kinase enzyme inhibitors. The scores in individual cell lines are given in Table. Our analysis showed that metformin was similar to fasudil with a score of 99.79 in MCF-7 cells and tolbutamide was similar to GSK-429286A with a score of 96.54 in HCC515. This analysis suggests that metformin and tolbutamide might be a potential Rho kinase inhibitor.

**Table:** Table. Comparison of metformin with fasudil and tolbutamide with GSK-429286A in the level of gene expression in cell lines using connectivity map.

	Summary	MCF-7	A375	A549	HCC515	HT29	PC3	HA1E	VCAP
Metformin vs. fasudil score	87.39	99.79	52.55	50.29	78.64	88.82	88.43	66.65	84.98
Tolbutamide vs. GSK-429286A score	53.62	11.41	80.54	44.22	96.54	85.40	-23.63	73.72	25.55

### 3.2. Effect of tolbutamide and metformin on cell proliferation

**Figure 1:**
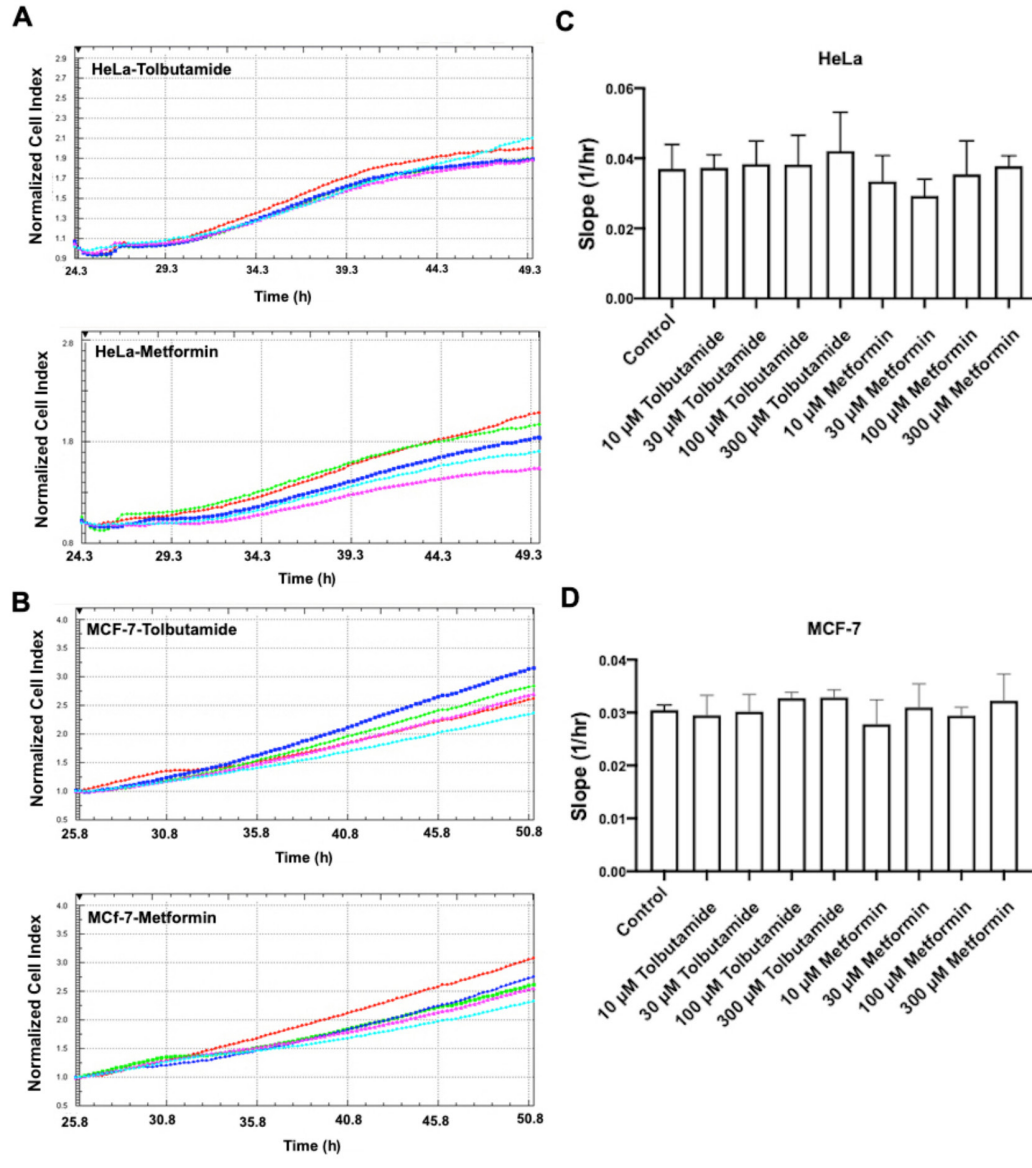
Monitoring of cell proliferation after the treatment of tolbutamide and metformin using the xCELLigence system. A. An original trace of tolbutamide- and metformin-treated HeLa cells recorded by RTCA DP instrument (In tolbutamide-treated trace, cyan curve: control, pink curve: 10 μM tolbutamide, blue curve: 30 μM tolbutamide, green curve: 100 μM tolbutamide, red curve: 300 μM tolbutamide) (In metformin-treated trace, red curve: control, cyan curve: 10 μM metformin, pink curve: 30 μM metformin, blue curve: 100 μM metformin, green curve: 300 μM metformin). B. An original trace of tolbutamide- and metformin-treated MCF-7 cells recorded by RTCA DP instrument (In tolbutamide-treated trace, red curve: control, pink curve: 10 μM tolbutamide, cyan curve: 30 μM tolbutamide, green curve: 100 μM tolbutamide, blue curve: 300 μM tolbutamide) (In metformin-treated trace, green curve: control, cyan curve: 10 μM metformin, pink curve: 30 μM metformin, blue curve: 100 μM metformin, red curve: 300 μM metformin). C. Slope of the curves in (A) as an index of the cell attachment rate. D. Slope of the curves in (B) as an index of the cell attachment rate. Data are means ± sem from 3 determinations.

To evaluate the effect of metformin and tolbutamide on cell proliferation, we treated cells with different concentrations of drugs and cells were monitored for 24 h. Our results showed that none of the tested concentrations of metformin and tolbutamide had no effect on cell proliferation (Figure 1). Thus we used nontoxic high concentrations of metformin and tolbutamide for their possible inhibitor effect on Rho kinase.

### 3.3. Effect of tolbutamide and metformin on morphology of cells

The possible effect of tolbutamide and metformin on cell morphology was assessed by a holographic microscope in HeLa and MCF-7 cells. Four morphological parameters, including cell area, cell volume, average thickness, and cell shape irregularities were calculated from the images obtained by a holographic microscope. One day after the cells were seeded, the cells were maintained with serum free DMEM for 24 h. Then the cells were exposed to tolbutamide and metformin, and the images were captured at 1 h and 24 h.

**Figure 2:**
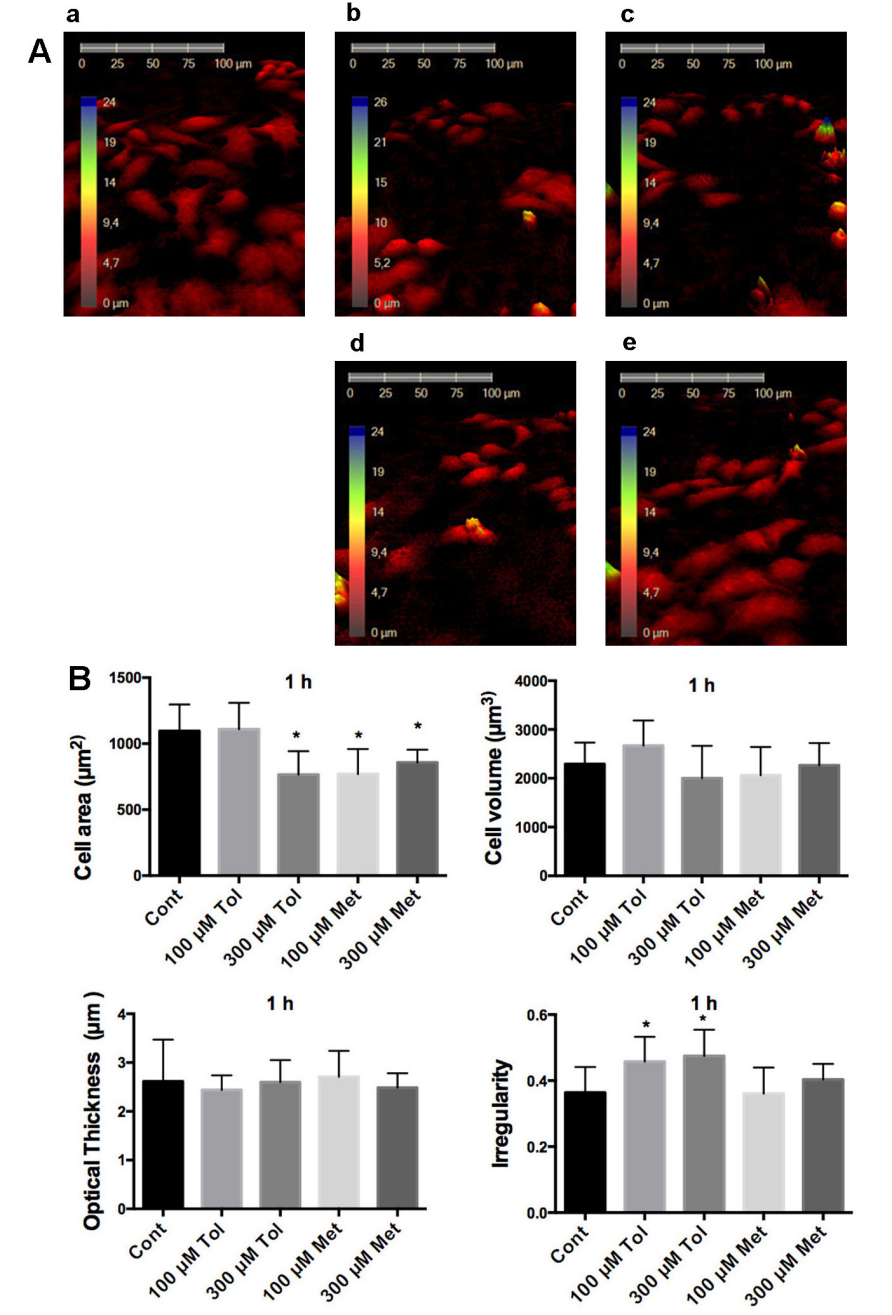
Investigation of HeLa cells morphology with Holomonitor M4 after 1 h drug incubation. A. Representative 3D pictures of HeLa cells treated with tolbutamide and metformin for 1 h. a: control, b: 100 μM tolbutamide, c: 100 μM metformin, d: 300 μM tolbutamide, e: 300 μM metformin. B. Statistical analysis of cell area, cell volume, average thickness, and irregular parameters in the form of cells. Cont: Control, 100 μM Tol: 100 μM Tolbutamide, 300 μM Tol: 300 μM Tolbutamide, 100 μM Met: 100 μM Metformin, 300 μM Met: 300 μM Metformin. * shows significant difference from the control group (one-way ANOVA, Tukey, P ≤ 0.05, n = 3).

**Figure 3:**
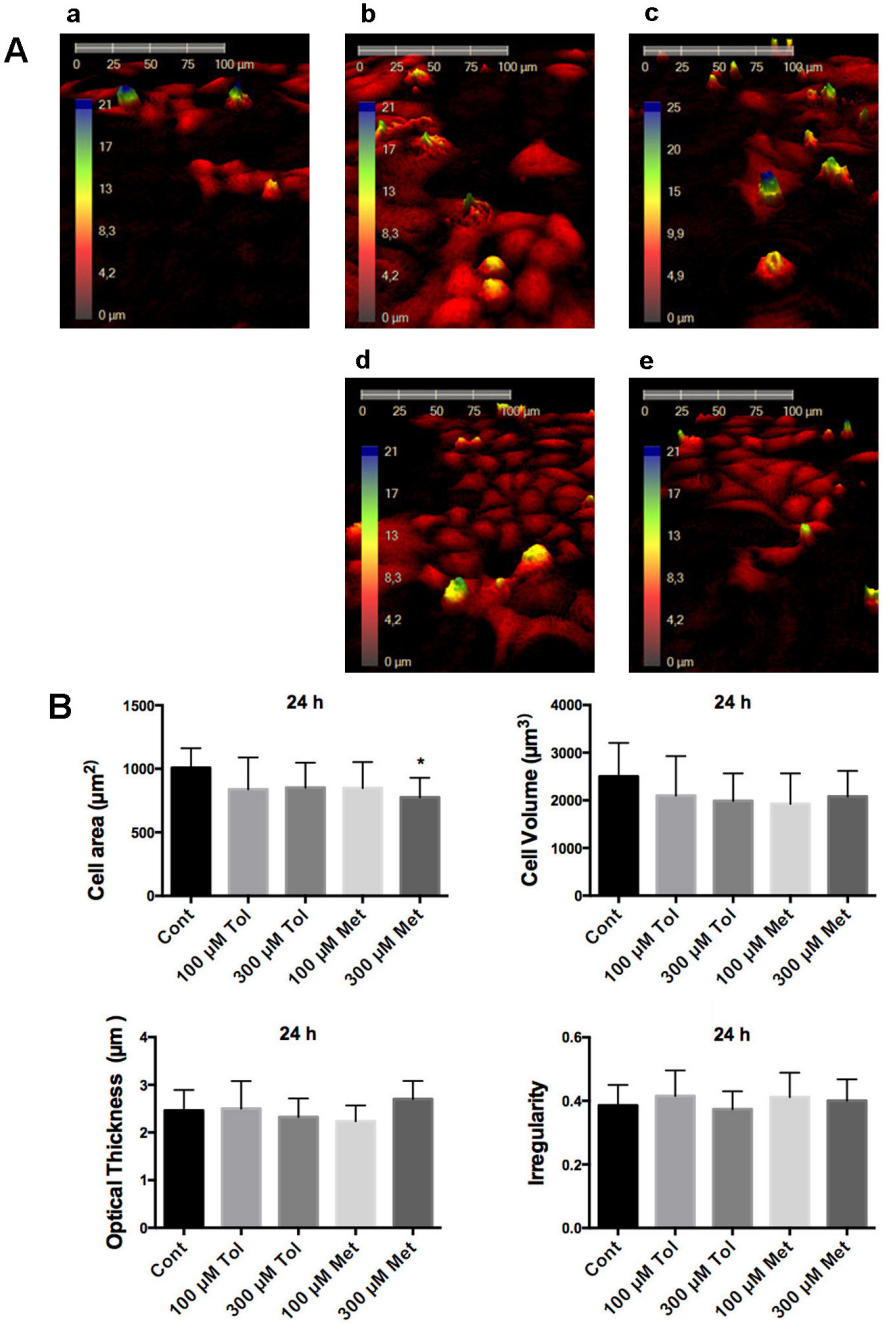
Investigation of HeLa cells morphology with HoloMonitor M4 after 24 h drug incubation. A. Representative 3D pictures of HeLa cells treated with tolbutamide and metformin for 24 h. a: control, b: 100 μM tolbutamide, c: 100 μM metformin, d: 300 μM tolbutamide, e: 300 μM metformin. B. Statistical analysis of cell area, cell volume, average thickness, and irregular parameters in the form of cells. Cont: Control, 100 μM Tol: 100 μM Tolbutamide, 300 μM Tol: 300 μM Tolbutamide, 100 μM Met: 100 μM Metformin, 300 μM Met: 300 μM Metformin. * shows significant difference from the control group (one-way ANOVA, Tukey, P ≤ 0.05, n = 3).

The measurements demonstrated that while 300 µM tolbutamide, 100 µM and 300 µM metformin decreased the cell area compared to the control group at 1 h (Figure 2), only incubation of 300 µM metformin for 24 h resulted in the decrease of the cell area in HeLa cells (Figure 3). In other morphological changes, only cell irregularity after the treatment of tolbutamide at 1h was increased significantly (Figure 2).

**Figure 4:**
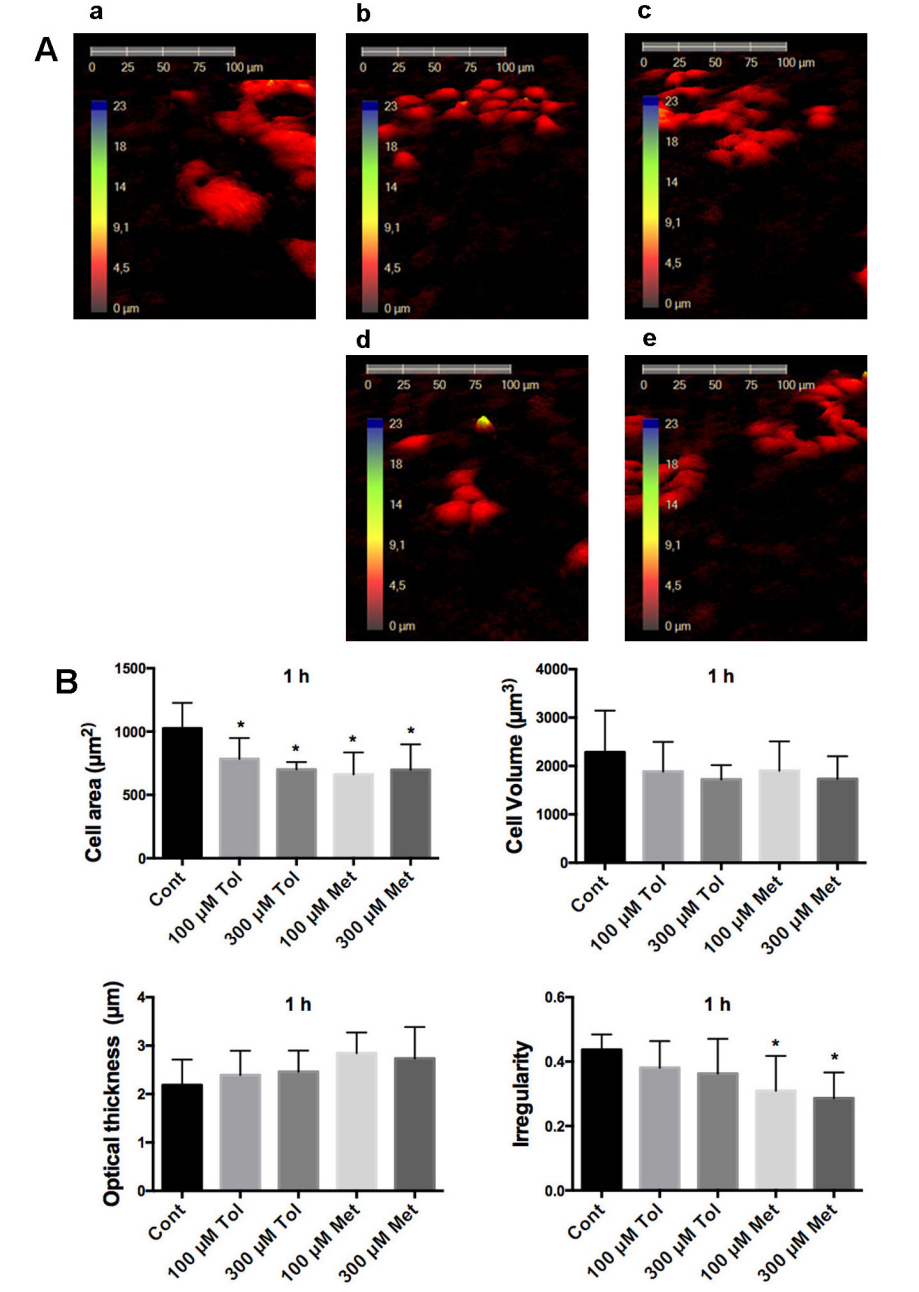
Investigation of MCF-7 cells morphology with HoloMonitor M4 after 1 h drug incubation. A. Representative 3D pictures of MCF-7 cells treated with tolbutamide and metformin for 1 h. a: control, b: 100 μM tolbutamide, c: 100 μM metformin, d: 300 μM tolbutamide, e: 300 μM metformin. B. Statistical analysis of cell area, cell volume, average thickness, and irregular parameters in the form of cells. Cont: Control, 100 μM Tol: 100 μM Tolbutamide, 300 μM Tol: 300 μM Tolbutamide, 100 μM Met: 100 μM Metformin, 300 μM Met: 300 μM Metformin. * shows significant difference from the control group (one-way ANOVA, Tukey, P ≤ 0.05, n = 3).

**Figure 5:**
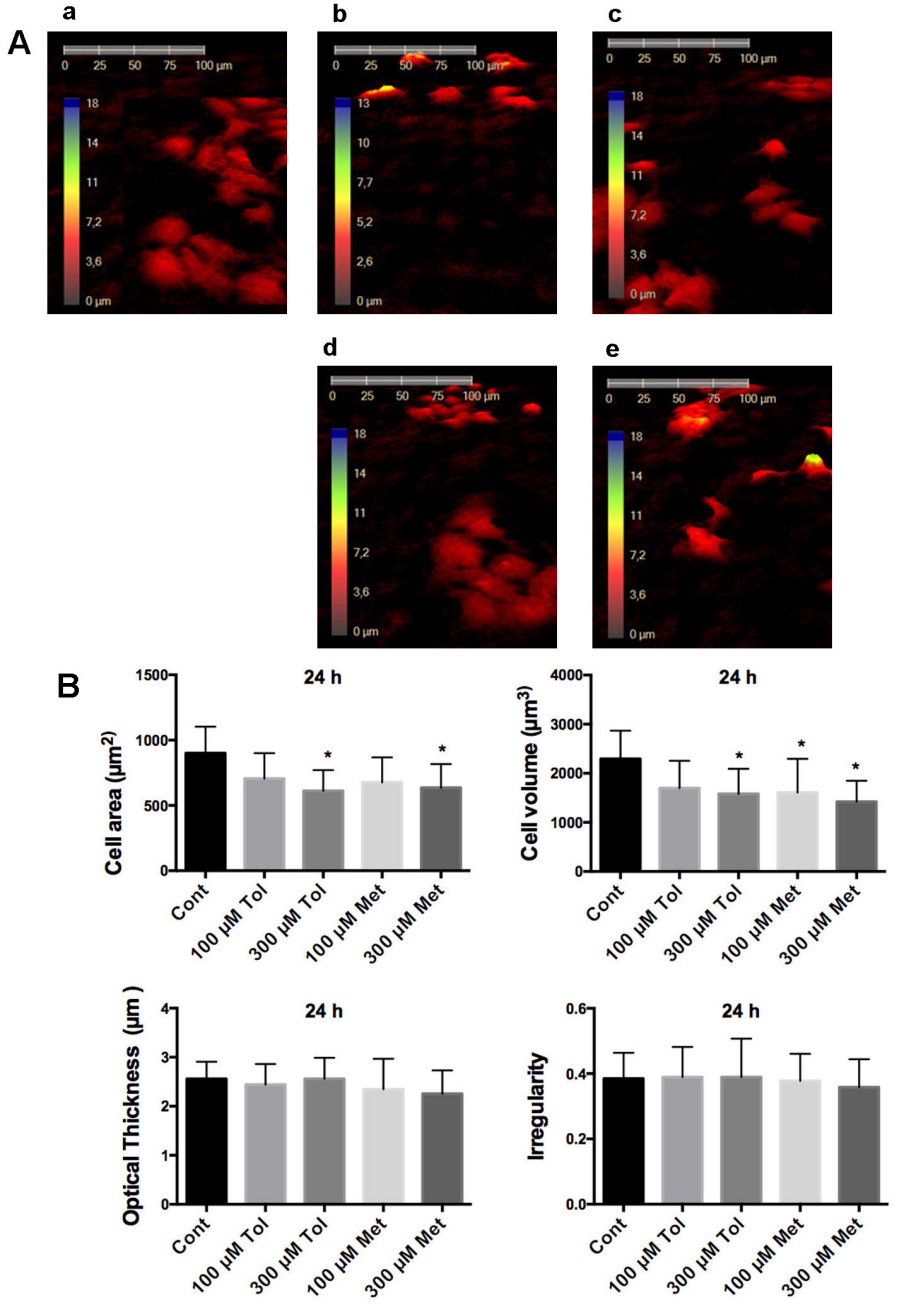
Investigation of MCF-7 cells morphology with HoloMonitor M4 after 24 h drug incubation. A. Representative 3D pictures of MCF-7 cells treated with tolbutamide and metformin for 24 h. a: control, b: 100 μM tolbutamide, c: 100 μM metformin, d: 300 μM tolbutamide, e: 300 μM metformin. B. Statistical analysis of cell area, cell volume, average thickness, and irregular parameters in the form of cells. Cont: Control, 100 μM Tol: 100 μM Tolbutamide, 300 μM Tol: 300 μM Tolbutamide, 100 μM Met: 100 μM Metformin, 300 μM Met: 300 μM Metformin. * shows significant difference from the control group (one-way ANOVA, Tukey, P ≤ 0.05, n = 3).

In MCF-7 cells, tolbutamide and metformin at all the concentrations at 1 h reduced the cell area significantly compared to the control (Figure 4). Additionally 300 µM tolbutamide and metformin decreased the cell area with 24-hour incubation (Figure 5). Interestingly, treatment of 300 µM tolbutamide, 100 µM and 300 µM metformin 300 for 24 h resulted in a significant reduction in cell volume in MCF-7 cells (Figure 5).

### 3.4. Effect of tolbutamide and metformin on the activation of ROCK

ROCK has many downstream effectors that are involved in cytoskeleton dynamics, such as MLC phosphatase, MLC, ERM and LIM kinase (Riento and Ridley, 2003). LIM kinase-described one of the substrates is cofilin family of actin-binding proteins, an actin-depolymerizing factor.  Once ROCK is activated, it phosphorylates concomitantly activates LIM kinase. Then LIM kinase phosphorylates cofilin at serine 3 and inactivates the protein. 

**Figure 6:**
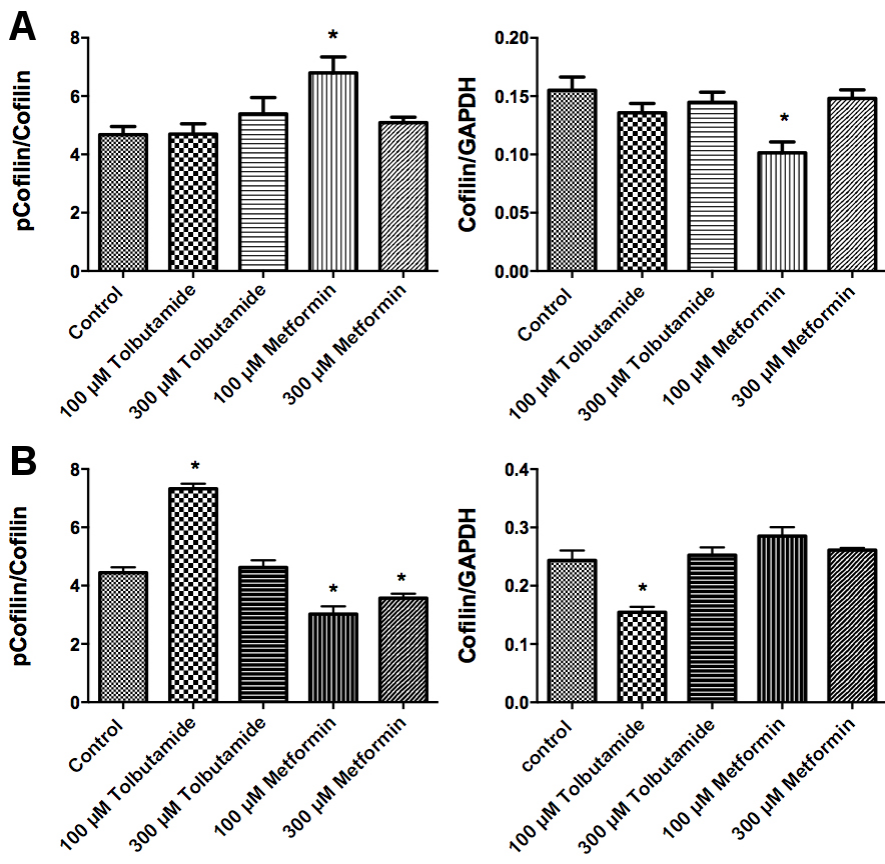
Determination of activation of Rho Kinase through the cofilin phosphorylation. A. Determination of cofilin phosphorylation in HeLa cells after 24-h drug incubation. B. Determination of cofilin phosphorylation in MCF-7 cells after 24-h drug incubation. * shows significant difference from the control group (one-way ANOVA, Tukey, P ≤ 0.05, n = 3).

To determine whether tolbutamide and metformin inhibit the activation of ROCK, we used phospho-cofilin (Ser3) colorimetric cell-based ELISA kit. HeLa and MCF-7 cells were seeded in 96-well plates. The next day, medium of the cells was replaced with serum free DMEM. After 24 h, the cells were incubated with an appropriate concentration of tolbutamide and metformin for 1 h. Then, according to the manufacturer’s instructions, ELISA kit was performed. We showed that 100 µM metformin increased the cofilin phosphorylation in HeLa cells (Figure 6A). However, while 100 µM tolbutamide increased the phosphorylation of cofilin, both concentrations of metformin decreased the phosphorylation of cofilin significantly in MCF-7 cells (Figure 6B) suggesting that metformin suppresses the activation of ROCK.

## 4. Discussion

Many drug molecules show their therapeutic effects through their specific targets. In addition to their primary targets, these drugs may also show an affinity for other unexpected off-target molecules (Tang et al., 2018). With the interaction of drugs and off-targets, the toxic effects of drugs can occur. Apart from the toxic effect, this undesirable interaction turns into an advantage in some cases. Defining off-target potencies of approved drugs enables the use of these drugs in various pathological conditions.

Apart from the effects of metformin in diabetes treatment, it also has beneficial effects in different physiopathologies. There are several data supporting that metformin is thought to be a promising agent for single use or chemotherapy combinations in cancer treatment. Metformin directly contributes to cancer treatment through upregulating miR-26 resulting in the inhibition of cell migration and invasion, and through the induction of apoptosis in some cancer cell lines (Li et al., 2012; Bao et al., 2012). These findings suggest that oral antidiabetics, especially metformin, may also be effective in different pathological conditions.

In this study, it was determined that metformin and tolbutamide, which are used in the treatment of type II diabetes, may cause inhibition of Rho kinase using connectivity map program. In order to confirm this data, MCF-7 and HeLa cells were exposed to these two small molecules, and the effects of these compounds on the morphological changes occurring as a result of Rho kinase activation and phosphorylation of cofilin were assessed.

ROCK has many substrates that are involved in the control of rearrangements of actin and microtubule cytoskeletons resulting in changes in cell morphology. Many studies indicated that inhibition of Rho kinase induced drastic alterations in cell shape. Previously we showed that pretreatment of Rock inhibitor, Y-27632, significantly reduced the increased cell area induced by cardiac glycosides in HeLa cells (Şimay et al., 2018). Zhang et al. (2011) demonstrated that treatment of both Y-27632 and HA-1077 resulted in changes in cell morphology inducing the formation of large numbers of protrusions in HOB and Saos-2 (Zhang et al., 2011). Therefore, metformin and tolbutamide were applied to the cells to examine the possible morphological change that drugs could induce through the inhibition of Rho kinase. We found that while metformin and tolbutamide reduced the cell area at 1h in HeLa and MCF-7 cells, only treatment of metformin resulted in a reduction of cell area at 24 h. This reduction in cell morphology pointed out that especially metformin may cause Rho kinase inhibition.

Activation of LIM kinase, one of the Rho kinase substrates, occurs as a result of Rho kinase activation. LIM kinases control cytoskeleton dynamics through the phosphorylation of the cofilin family proteins. When cofilin is phosphorylated by LIMK, it is inactive. To examine whether tolbutamide and metformin would cause inhibition of Rho kinase and whether the reduction in cell area would be accompanied by Rho kinase, the phosphorylation of cofilin was evaluated. In MCF-7 cell, metformin treatment significantly reduced cofilin phosphorylation compared to the control group indicating that metformin inhibits ROCK activation. These results suggested that metformin can inhibit Rho kinase in a cell type-dependent manner.

Although our findings suggest that metformin might be a ROCK inhibitor, whether this inhibition occurs directly from the interaction of ROCK and metformin directly has not been shown.

There are major signaling pathways that regulate cytoskeletal properties thereby cell morphology such as PI3 kinase‒Akt and Rho‒ROCK pathway (Amano et al., 2010; Jo et al., 2014; Moujaber and Stochaj, 2020). The reduction in cell area after the treatment of MCF-7 cells with tolbutamide for 24 h gave the clue that tolbutamide might inhibit ROCK. However, when we evaluated the activation of Rho kinase after tolbutamide treatment, we showed that tolbutamide had no effect on ROCK activation suggesting that the decrease in cell area occurs in a Rho kinase-independent manner. These data suggest that tolbutamide might affect cell morphology through another pathway involved in actin cytoskeleton organization such as the PI3 kinase‒Akt pathway. 

In conclusion, our results enabled metformin to be identified as a new and approved inhibitor of ROCK. It also suggested that especially metformin that is approved by FDA might be used for the treatment in various pathological conditions in where Rho kinase is involved such as asthma, cancer, erectile dysfunction, glaucoma.
